# Preparation of Cyano-Substituted Tetraphenylethylene Derivatives and Their Applications in Solution-Processable OLEDs

**DOI:** 10.3390/molecules23010190

**Published:** 2018-01-17

**Authors:** Xiaoyi Sun, Lele Zhao, Xiao Han, Hui Liu, Yu Gao, Yanchun Tao, Haiquan Zhang, Bing Yang, Ping Lu

**Affiliations:** 1 State Key Laboratory of Supramolecular Structure and Materials, Jilin University, 2699 Qianjin Avenue, Changchun 130012, China; xysun15@mails.jlu.edu.cn (X.S.); challenge_llzhao@163.com (L.Z.); slan521241@126.com (X.H.); liuhui17@mails.jlu.edu.cn (H.L.); iambird83317035@126.com (Y.G.); taoyc@jlu.edu.cn (Y.T.); yangbing@jlu.edu.cn (B.Y.); 2 State Key Laboratory of Metastable Materials Science and Technology, Yanshan University, Qinhuangdao 066004, China; hqzhang@ysu.edu.cn

**Keywords:** diphenylfumaronitrile, fluorene, AIE, solution-processability, OLED

## Abstract

Creation of organic luminescent materials with high solid-state efficiency is of vital importance for their applications in optoelectronic fields. Here, a series of AIE luminogens (*AIE* gens), (*Z*)-2,3-bis(4-(9,9-bis(6-(9*H*-carbazol-9-yl)hexyl)-9*H*-fluoren-2-yl)phenyl)-3-phenylacrylonitrile (SFC), and 2,3-bis(4-(9,9-bis(6-(9*H*-carbazol-9-yl)hexyl)-9*H*-fluoren-2-yl)phenyl)fumaronitrile (DFC), utilizing 2,3,3-triphenylacrylonitrile and 2,3-diphenylfumaronitrile as respective centers, are designed and synthesized by Suzuki coupling reactions with high yields. The *cis*- and *trans*-isomers of DFC are also successfully obtained. All of them are thermally stable and show good solubility in common organic solvents. They all emit weakly in solution, but become strong emitters when fabricated into solid films. It is found introduction of one additional cyano group in DFC induced a big red-shift in solid-state emission, owing to its high electron-withdrawing ability. The *cis*- and *trans*-DFC show similar photophysical and Cyclic voltammogram (CV) behaviors. Non-doped solution-processed organic light-emitting diodes (OLEDs) using the three compounds as light-emitting layers are fabricated. SFC gives the best device performance with a maximum luminance of 5201 cd m^−2^, a maximum current efficiency of 3.67 cd A^−1^ and a maximum external quantum efficiencies (EQE) of 1.37%. Red-shifted EL spectra are observed for *cis*- and *trans*-DFC-based device, and the OLED using *trans*-DFC as active layer exhibits better performance, which might derive from their different conformation in film state.

## 1. Introduction

Tremendous progress has been achieved in organic luminescent materials over the past decades, due to their potential applications in optoelectronic fields such as organic light emitting diodes (OLEDs) [1,2,3,4], organic field-effect transistors (OFETs) [5,6,7,8], non-linear optics [9], and chemical sensors [10], etc. Materials with good solubility are highly desirable, because they are regarded as the most promising candidates for the fabrication of low-cost optoelectronic devices [11]. The high quantum efficiency in the solid-state is of vital importance, since the luminescent materials are applied in film state in most cases. However, many π-systems become weakly emissive when they are in solid or aggregation states, despite being strongly emissive in dilute solutions. This effect is termed as aggregation caused emission quenching (ACQ) [12]. In this view, a notable example is aggregation-induced emission (AIE), first reported by Luo et al. [13], which refers to the phenomenon of materials that are non-emissive in solution, and emit intensely in the aggregate state, owing to the restriction of the intramolecular rotation process and the suppression of intermolecular interactions. It provides a unique way to endow materials with high photoluminescent quantum efficiency in aggregated states. Tetraphenylethylene (TPE) represents the most investigated chromophore with a typical AIE property, and its derivatives have been widely utilized in OLEDs, two-photon absorption, fluorescence detection of 2,4,6-trinitrotoluene (TNT), biological sensors, and so on [14,15,16,17].

Recently, we have developed a new kind of TPE derivative with high quantum yield in solid state, diphenylfumaronitrile, which has shown the potential to undergo a reverse intersystem crossing process along the high-lying charge transfer (CT) channel, to reach a compromise of local emission (LE) and CT states. Its derivative, 2,3-bis(4′-(diphenylamino)-[1,1′-biphenyl]-4-yl)fumaronitrile(TPATCN), which exerts the advantages of the relatively large dipole moment of the charge transfer state and a certain degree of orbital overlap of the local excited state, breaks through the 25% upper limit of singlet ratios in OLEDs [18]. To further expand the application of diphenylfumaronitrile group in solution-processable OLEDs, herein, it was attached with 9,9-dihexylfluorene to increase the solubility in common organic solvent. We also applied 2,3-bis(4-bromophenyl)-3-phenylacrylonitrile as a central unit, to investigate the effect of number of cyano groups on the optical property of materials. Carbazole is a commonly utilized organic luminescent material with good hole-injection ability and high thermal stability [19,20]. Two carbazole units were appended at the end of 9,9-dihexyl side chain, because the cyano functionality allows for great electron delocalization, due to its high electron-withdrawing ability. Guided by this design principle, two D-A-D type fluorescent compounds, (*Z*)-2,3-bis(4-(9,9-bis(6-(9*H*-carbazol-9-yl)hexyl)-9*H*-fluoren-2-yl)phenyl)-3-phenylacrylonitrile (SFC), and 2,3-bis(4-(9,9-bis(6-(9*H*-carbazol-9-yl)hexyl)-9*H*-fluoren-2-yl)phenyl)fumaronitrile (DFC), are successfully obtained. In addition, the pure *cis*- and *trans*-isomers of DFC are successfully separated and purified. All the three compounds are found to possess high photoluminescence quantum efficiencies, good thermal stability, and high morphological stability. Using the spin-coating films as the active layers, OLEDs using SFC film as active layer are achieved with a maximum luminance of 5201 cd m^−2^, and a maximum LE of 3.67 cd A^−1^. *Trans*-DFC-based device showed a much red-shifted electroluminescence with a maximum luminance of 4025 cd m^−2^, and a maximum LE of 1.96 cd A^−1^, which is also better than the performance of *cis*-DFC-based device.

## 2. Results and Discussion

### 2.1. Synthesis and Characterization

**Scheme 1:**
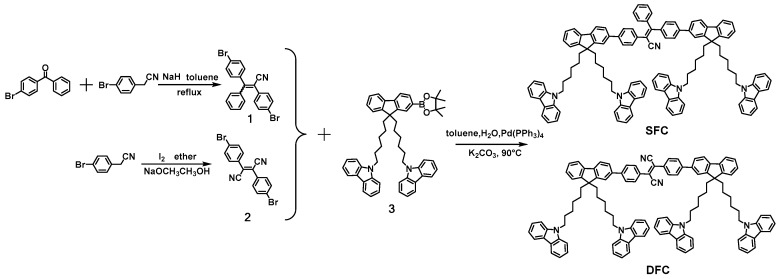
Synthetic route to (*Z*)-2,3-bis(4-(9,9-bis(6-(9*H*-carbazol-9-yl)hexyl)-9*H*-fluoren-2-yl)phenyl)-3-phenylacrylonitrile (SFC) and 2,3-bis(4-(9,9-bis(6-(9*H*-carbazol-9-yl)hexyl)-9*H*-fluoren-2-yl)phenyl)fumaronitrile (DFC).

The chemical structures of SFC and DFC are shown in Scheme 1. 9,9′-((2-(4,4,5,5-Tetramethyl-1,3,2-dioxaborolan-2-yl)-9*H*-fluorene-9,9-diyl)bis(hexane-6,1-diyl))bis(9*H*-carbazole) (**3**) was synthesized starting from commercially available fluorene and carbazole through a three step reaction, according to reported literature [21]. SFC and *trans*-DFC were prepared through Suzuki coupling reaction between monomer 3 and 2,3-bis(4-bromophenyl)-3-phenylacrylonitrile (**1**) or diphenylfumaronitrile (**2**), respectively. The photoisomerization process of *trans*-DFC could occur after exposure to UV light for some time, and the pure *cis*-DFC was also successfully separated. SFC, *trans*-DFC, and *cis*-DFC were fully characterized by ^1^H-NMR, MS, and elemental analysis, and corresponded well with their expected structure (Supplementary Materials). They all showed high solubility (>10 mg mL^−1^) in common organic solvents, such as toluene, tetrahydrofuran (THF), dichloromethane (DCM), and chloroform, which fulfill the requirement for solution-processability.

### 2.2. Thermal Stablity

**Figure 1:**
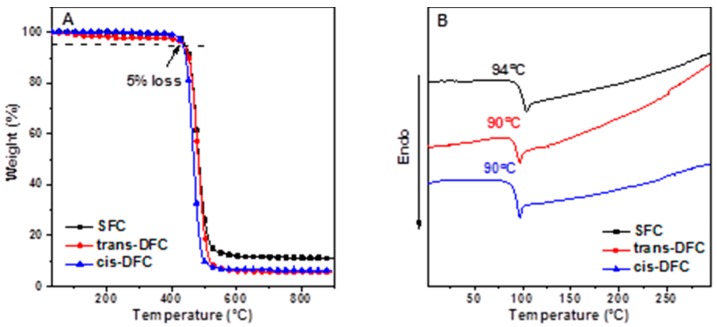
Thermogravimetric analysis (TGA) (**A**) anddifferential scanning calorimetry (DSC) (**B**) graphs of SFC, *trans*-DFC and *cis*-DFC.

Thermogravimetric analysis (TGA) and differential scanning calorimetry (DSC) were carried out under a nitrogen atmosphere to investigate their thermal properties. The DSC results revealed that SFC, *cis*-DFC, and *trans*-DFC showed very similar *T*_g_ values of 94 °C, 90 °C, and 90 °C, respectively (Table 1). The relatively low *T*_g_ values might result from the two long alkyl side chains appended on fluorene, which endowed them with good flexibility. They all exhibited the highest decomposition temperature (T_d_, corresponding to 5% weight loss) of 442 °C, 438 °C, and 435 °C (Figure 1). The good thermal stability is very important for their applications in optoelectronic fields.

### 2.3. Morphological Stablity

**Figure 2:**
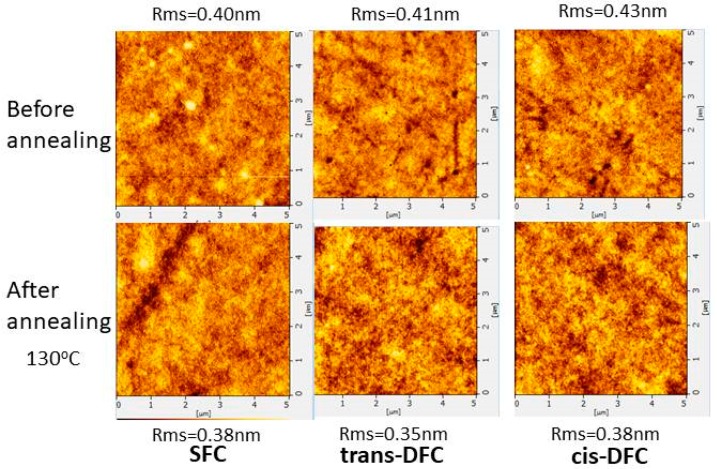
AFM images of SFC, *trans*-DFC and *cis*-DFC on indium tin oxide (ITO) at room temperature (**up**) and after annealing at 130 °C for 30 min (**down**).

The morphological stability of the solution-processed films of SFC, *trans*-DFC, and *cis*-DFC were estimated by atomic force microscopy (AFM). The films were spin-coated from chlorobenzene solution with concentration of 10 mg mL^−1^. Then, the samples were annealed at 130 °C for 30 min. The AFM images are given in Figure 2. All the spin-coated films of SFC, *cis*-DFC, and *trans*-DFC showed smooth surfaces with very small root-means-square (RMS) values that ranged from 0.40 to 0.43 nm. The results suggested that introducing flexible alkyl chains could provide homogeneous morphologies of resulting materials. All the films remained almost unchanged after thermal annealing, with RMS values of 0.35–0.38 nm, indicating their possibility in keeping uniform films throughout the device fabrication process.

### 2.4. Photophysical Property

**Figure 3:**
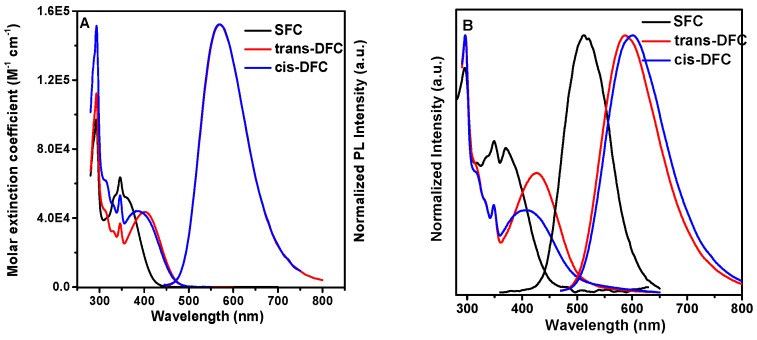
(**A**) The UV–vis and PL spectra in diluted THF solution of SFC, *trans*-DFC and *cis*-DFC (concentration: 1 × 10^−5^ mol L^−1^); (**B**) The UV–vis and PL spectra in films of SFC, *trans*-DFC, and *cis*-DFC.

The UV–vis absorption and PL spectra of SFC, *trans*-DFC and *cis*-DFC in dilute THF solution and thin films are provided in Figure 3. The similar absorption peak at 291 nm and 346 nm were both observed in the spectra, which were caused by the π–π* transition of the benzene rings and fluorene, respectively. There also existed an additional absorption peak at a longer wavelength of 400 nm and 387 nm in *trans*-DFC and *cis*-DFC, as compared with SFC. The absorption onset of them (480 nm) also showed a big red-shift compared to that of SFC (420 nm). Since the conjugation length between SFC and DFC are quite similar, the new absorption peak in red-shifted region of DFC is possibly from the stronger CT transition caused by the introduction of one more cyano group in the central part. The absorption onset of SFC, *trans*-DFC and *cis*-DFC in film state are 447 nm, 496 nm, and 486 nm, and the corresponding optical gaps were thus calculated to be 2.77 eV, 2.50 eV, and 2.55 eV, respectively.

**Figure 4:**
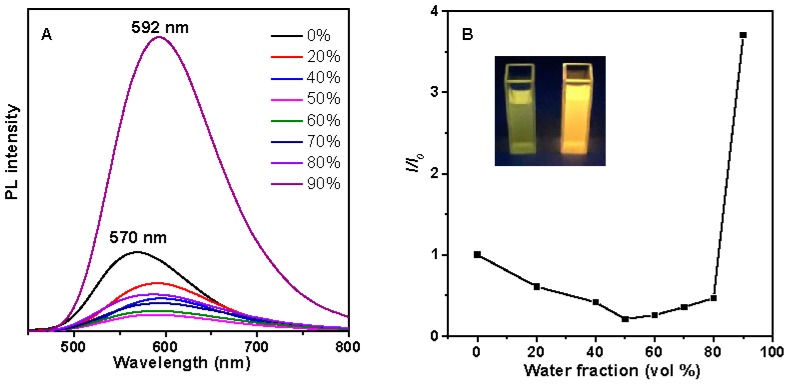
(**A**) PL spectra of *trans*-DFC in THF–water mixtures with different water fractions (*f*_w_); (**B**) Plot of the relative PL intensity (*I/I*_0_) versus the compositions of THF–water mixtures of *trans*-DFC. *I*_0_ = emission intensity in pure THF solution. Inset in (**B**) are the fluorescent photographs at *f*_w_ = 0 and 95% taken under a 365 nm UV lamp.

SFC was non-emissive with a very low quantum efficiency of 0.08% using sulfate quinone as a reference in dilute solution, because the molecules totally dissolved in the mixture. It began to emit intensively when the water fraction (*f*_w_) reached 60%, which is a typical AIE characteristic (Supplementary Materials Figure S1). The *cis*- and *trans*-isomer of DFC displayed very similar photophysical behaviors, although they showed different apparent colors. Take *trans*-DFC as an example, a weak emission was observed in dilute THF with a quantum efficiency of 7.4%. The emission intensity was gradually decreased as the water fraction was continuously increased, accompanied by a red-shifted emission peak. This weak fluorescence in pure THF might be related to the twisted configuration in the conjugated backbone, and the corresponding intramolecular interactions, which induced the CT effects, and resulted in a relatively low efficiency in THF. When *f*_w_ continued to increase over 60%, the emission peak maximum appeared as an unusual blue-shift, and the intensity was increased quickly, indicating that the molecules aggregation began to occur, and the fluorescence was further enhanced with the increase of the water fraction, confirming its aggregation-induced emission enhancement (AIEE)-active nature (Figure 4, Figures S6 and S7) [22]. The corresponding intramolecular motion is a conventional mechanism for these AIEgens. *cis*-DFC also displayed the similar photoluminescence behavior (Supplementary Materials Figure S2). The photoisomerization process could be clearly observed from the ^1^H-NMR spectra by monitoring the solution under UV light. (Supplementary Materials Figure S3) The results indicated the photochemical process was also an important reason for the low efficiency in THF. 

To get a better insight into the ground state and the excited state properties, the salvation effects on photoluminescence (PL) were measured in various solvents ranging from nonpolar solvent *n*-hexane to highly polar solvent acetonitrile. The CT character of the excited state was further evidenced by the red shift of the emission maximum upon increasing solvent polarity (Supplementary Materials Figure S5). For instance, the emission peak of *trans*-DFC shifted from 542 to 590 nm when measured in nonpolar hexane and methylene dichloride, respectively. Correspondingly, the photoluminescent quantum yields were continuously decreased from 8.4 to 0.7%, which is a typical photophysical phenomenon that arises from intramolecular charge transfer [23].

**Figure 5:**
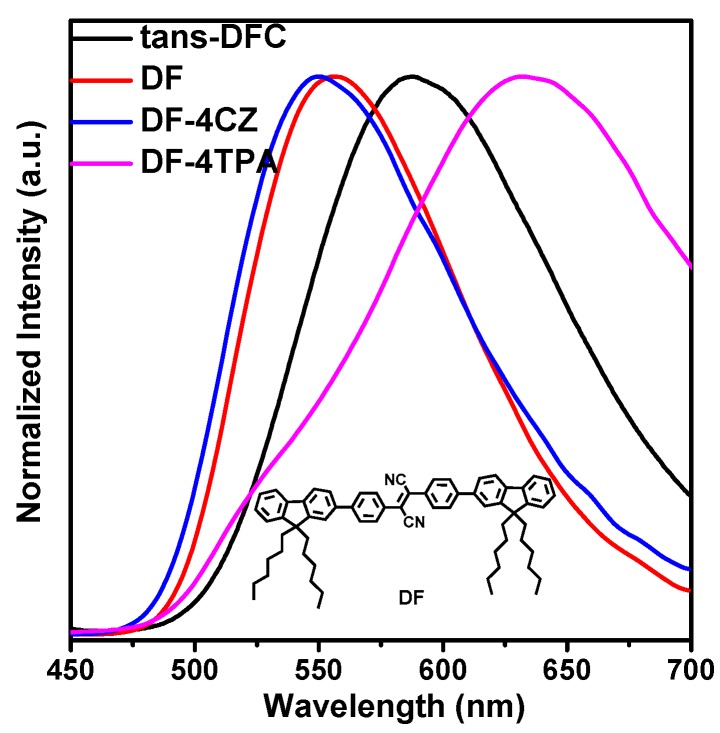
The PL spectra in films of *trans*-DFC, 2,3-bis(4-(9,9-dihexyl-9*H*-fluoren-2-yl)phenyl)fumaronitrile (DF), and DF dispersed in carbazole with a mole ratio of 1:4. Inset: the structure of DF. (CZ = carbazole; TPA = triphenylamine).

The films of the SFC exhibited strong green emission peaking at 513 nm. *Trans*-DFC and *cis*-DFC exhibited strong red-shifted emission peaking at 587 nm and 600 nm, respectively. Compared with the compound TPE-DFCz without cyano group which was reported previously [24], the emission in film state was red-shifted 26 nm in SFC, 100 nm in *trans*-DFC, and 113 nm in *cis*-DFC. To further clarify the origin of the red-shift, we also synthesized the model compound 2,3-bis(4-(9,9-dihexyl-9*H*-fluoren-2-yl)phenyl)fumaronitrile (DF) without carbazole units (inset in Figure 5). In the doped film of DF dispersed in carbazole with a mole ratio of 1:4, it showed a similar emission with that of DF (Figure 5), indicating very weak intramolecular charge transfer from carbazole to DF, since carbazole is a relatively weak donor. If the DF was dispersed in triphenylamine with the same mole ratio, the emission peak of the doped film was red-shifted to 633 nm. It can be seen that the intermolecular charge transfer could occur if it was doped with relatively stronger donor of triphenylamine. Thus, the red-shift from SFC to DFC is not only caused by the intramolecular charge transfer from the fluorene group to the central nucleus, but also from the carbazole to the central nucleus because the flexible dihexyl side chain can be folded to some extent. SFC powder exhibited a photoluminescent quantum yield (PLQY) of 46%. The powder PLQY of *trans*-DFC was 54.5%, which was comparable to that of *cis*-DFC (55.4%).

### 2.5. Electrochemical Properties

**Figure 6:**
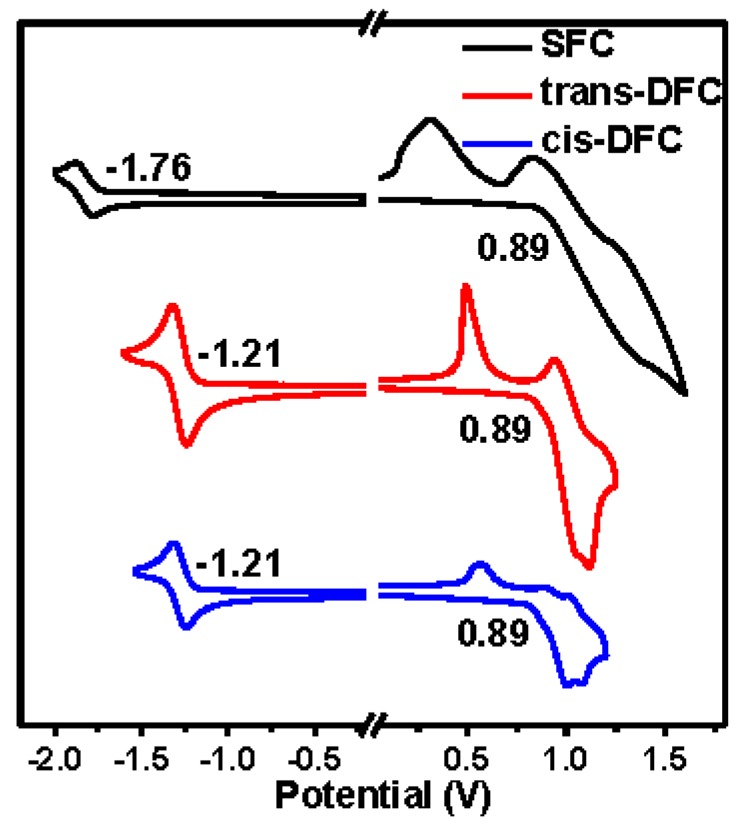
Cyclic voltamogram of SFC, *trans*-DFC, and *cis*-DFC measured in dry CH_2_Cl_2_ (oxidation) and DMF (reduction) with 0.1 M Bu_4_NPF_6_ as supporting electrolyte, at a scan rate of 100 mV s^−1^.

In the cyclic voltammetry measurement, the oxidation and reduction potentials of SFC were found to be at 0.89 V and −1.76 V against ferrocenium/ferrocene (fc^+^/fc) redox couple (Figure 6), corresponding to a HOMO level of −5.48 eV and a LUMO level of −2.97 eV, respectively. Similar HOMO and LUMO levels were both obtained for *trans*- and *cis*-DFC. Their HOMO levels were both calculated to be −5.48 eV, and the LUMO levels were both −3.51 eV. Their HOMO levels were the same as that of SFC, which were all determined by the carbazole units [25]. The introduction of an additional cyano group in DFC has induced a 0.54 eV decrease in LUMO energy level.

### 2.6. Electroluminescence Properties

**Figure 7:**
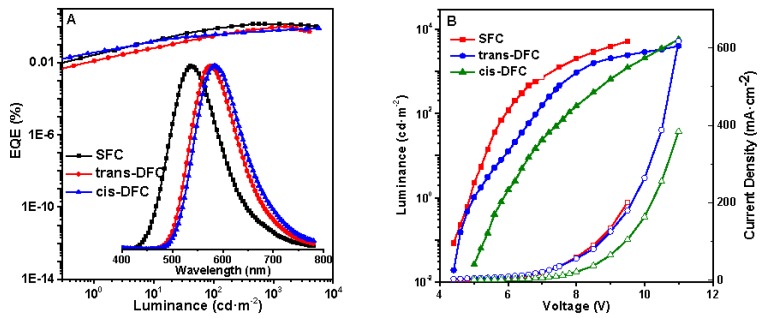
(**A**) EQE of SFC, *trans*-DFC, and *cis*-DFC and their normalized EL spectra; (**B**) Luminance–voltage–current density characteristics of multilayer EL devices of SFC, *trans*-DFC, and *cis*-DFC.

The high solid fluorescence quantum yield and good solubility of the present molecules encouraged us to investigate their applications in low-cost OLEDs. The non-doped solution-processed OLEDs were fabricated with the configuration of indium tin oxide (ITO)/poly(3,4-ethylenedioxythiophene):poly(styrenesulfonate) (PEDOT-PSS) (40 nm)/EML (60 nm)/TmPyPB (25 nm)/LiF (0.5 nm)/Al (100 nm), in which ITO-coated glass was used as both the substrate and anode, PEDOT-PSS as a hole-transporting layer, TmPyPB as an electron-transporting layer, and LiF as the modificatory layer of the cathode Al. The characterization data for the three devices were summarized in Table 2. All the three compounds exhibited identical EL spectra similar to their respective PL spectra of the spin-coated films. As shown in Figure 7, the maximum peaks in the EL spectra were observed at 536 nm, 576 nm, and 584 nm for SFC, *trans*-DFC, and *cis*-DFC, respectively, suggesting that the EL emissions originated from the active layers. Furthermore, the three devices all showed very stable emission at voltages from 7 to 14 V (Supplementary Materials Figure S4) arising from their good thermal and morphological stabilities. SFC showed green emission with a maximum current efficiency (CE) of 3.67 cd/A, a maximum luminance of 5201 cd m^−2^, and an external quantum efficiency (EQE) of 1.37%. The *trans*-DFC showed a 40 nm red-shifted electroluminescence spectrum with a maximum luminance of 4025 cd m^−2^, a maximum CE of 1.97 cd/A, and an EQE of 0.99%, which was a little higher than *cis*-DFC in terms of efficiencies (5716 cd m^−2^, 1.49 cd/A, 0.79%). This might be related the more planar conformation of *trans*-isomer, which was more beneficial for the carrier transportation than the *cis*-one (Table 2).

## 3. Materials and Methods

### 3.1. Instrumentation

All the reagents were purchased from Acros, J&K and Aldrich Chemical Co. (Shanghai, China) and used as received. THF was dried and purified by fractional distillation over sodium in the presence of benzophenone. The electrochemical properties (oxidation and reduction potentials) were carried out via cyclic voltammetry (CV) measurements by using a standard one-compartment, three-electrode electrochemical cell given by a BAS 100 B/W electrochemical analyzer. 0.1 M tetrabutylammoniumhexafluorophosphate (TBAPF_6_) in anhydrous dimethylformamide (DMF) or anhydrous dichloromethane was used as the electrolyte for negative or positive scan. A glass–carbon disk electrode was used as the working electrode, a Pt wire as the counter electrode, and Ag/Ag^+^ as the reference electrode, together with ferrocene as the internal standard at a scan rate of 100 mV s^−1^. The ^1^H-NMR were recorded on a Bruker AVANCE 500 spectrometers at 500 MHz at 298 K using CDCl_3_ as solvent and tetramethylsilane (TMS) as internal standard. All of the compounds were characterized by a Flash EA 1112, CHNS elemental analysis instrument. The MALDI-TOF-MS mass spectra were recorded using an AXIMA-CFRTM plus instrument. UV–vis absorption spectra were measured on Shimadzu UV-3100PC spectrophotometer, and fluorescence recorded by using a RF-5301PC. Differential scanning calorimetry (DSC) analysis was carried out using a TAQ100 instrument at 10 °C min^−1^ while flushing with nitrogen. Thermal gravimetric analysis (TGA) was recorded on a Perkin Elmer thermal analysis system at a heating rate of 10 °C min^−1^ and a nitrogen flow rate of 80 mL min^−1^. The atomic force microscopy (AFM) images were recorded by a Seiko SPA 300 in contact mode (AFM mode).

### 3.2. Device Fabrication

The ITO-coated glass substrates were cleaned in an ultrasonic bath with purified water, isopropylalcohol, acetone, toluene, acetone, isopropylalcohol, respectively, and cleaned for 30 min by exposure to UV-ozone. Subsequently, a 40 nm-thick poly(3,4-ethylenedioxythiophene):poly(styrenesulfonate) (PEDOT-PSS, Batron-P4083, Bayer AG) film was spin-coated at 4000 rpm for 60 s on top of the ITO and then baked at 120 °C for 30 min. Then, solutions of SFC and DFC in chlorobenzene (10 mM mL^−1^) were spin-coated on (2000 rpm for 30 s) PEDOT-PSS as the emissive layer (EML). After annealing at 130 °C for 30 min, the thickness of the EML was about 60 nm. Then 25 nm-thick TmPyPb, as an electron-transporting layer, was thermally deposited onto the emitting layers under vacuum with a back pressure of 10^−5^ Pa. Finally, LiF buffer layer and an Al cathode were deposited onto TmPyPb. The luminance–current characteristics of devices were measured using a PR650 spectroscan spectrometer, and current–voltage characteristics were recorded by a Keithley 2400 sourcemeter. All of procedure was performed at room temperature under ambient conditions.

### 3.3. Experimental Section

*Synthesis of 2,3-bis(4-bromophenyl)-3-phenylacrylonitrile (M1)*: (4-bromophenyl)(phenyl)methanone (3 g, 11.5 mmol) and 60% NaH (1.8 g, 46 mmol) were added into a 250 mL two-necked round-bottomed flask. The flask was degassed and flushed with dry nitrogen, after which toluene (100 mL) was injected. The mixture was heated to 80°C, then toluene solution (50 mL) of 2-(4-bromophenyl)acetonitrile (2.25 g, 16 mmol) was added slowly. After refluxing overnight, the reaction was quenched with water and extracted with DCM. The organic layer was washed with brine and dried over anhydrous sodium sulfate. After filtration and solvent evaporation, the residue was purified by silica gel column chromatography, using petroleum ether and DCM as eluent. M1 (1.0 g) was obtained as a light yellow solid. Yield: 20%.

*Synthesis of 2,3-bis(4-bromophenyl)fumaronitrile (M2)*: 2-(4-bromophenyl)acetonitrile (1 g, 5.1 mmol) and I_2_ (1.3 g, 5.1 mmol) were dissolved in diethyl ether under nitrogen. The system temperature was stabilized at −78 °C, and sodium methylate (1.7 g, 10.71 mmol) was added to the reaction system and stirred for 30 min. Then, the reaction mixture was transferred to ice water and stirred for further 4 h. Thereafter, a small amount of 3% HCl (aq) was added to quench the reaction, and then M2 was isolated from the reaction solution by filtration. Yield: 70%.

*Synthesis of (Z)-2,3-bis(4-(9,9-bis(6-(9H-carbazol-9-yl)hexyl)-9H-fluoren-2-yl)phenyl)-3-phenylacrylonitrile (SFC)*: 2,3-bis(4-bromophenyl)-3-phenylacrylonitrile (439 mg, 1 mmol), 2-9,9′-((2-(4,4,5,5-tetramethyl-1,3,2-dioxaborolan-2-yl)-9*H*-fluorene-9,9-diyl)bis(hexane-6,1-diyl))bis(9*H*-carbazole) (1.74 g, 2.2 mmol), toluene (30 mL), Na_2_CO_3_ (2 M,10 mL), and THF (15 mL) were put into a 100 mL two-necked round-bottom flask equipped with a reflux condenser. Pd(PPh_3_)_4_ (80 mg) was added under nitrogen protection. The mixture was refluxed for 24 h. After that, the mixture was cooled to room temperature and water was added to quench the reaction. The organic layer was washed and extracted with dichloromethane. Compound SFC (300 mg) was then isolated by column chromatography using a mixture of petroleum and dichloromethane (4:1) as eluent. Yield: 50%. ^1^H-NMR (500 MHz, CDCl_3_) δ 8.14–8.04 (m, 7H), 7.77 (d, *J* = 7.9 Hz, 1H), 7.75–7.68 (m, 4H), 7.63 (d, *J* = 7.9 Hz, 1H), 7.57 (t, *J* = 9.0 Hz, 3H), 7.55–7.50 (m, 3H), 7.48 (d, *J* = 8.2 Hz, 3H), 7.42 (dd, *J* = 16.3, 8.1 Hz, 9H), 7.35 (d, *J* = 7.3 Hz, 3H), 7.31 (dd, *J* = 7.0, 4.1 Hz, 6H), 7.26 (d, *J* = 7.6 Hz, 3H), 7.21 (dd, *J* = 12.4, 7.4 Hz, 8H), 7.16 (d, *J* = 7.2 Hz, 2H), 7.12–7.07 (m, 1H), 4.18 (dd, *J* = 11.7, 7.1 Hz, 9H), 1.95 (dd, *J* = 16.9, 8.6 Hz, 9H), 1.70 (dd, *J* = 14.2, 7.2 Hz, 8H), 1.29–0.92 (m, 17H), 0.62 (d, *J* = 7.5 Hz, 10H).MALDI-TOF MS (mass *m/z*):1608.5 [M+]. Anal. calcd. for C_119_H_107_N_5_: C 88.93, H 6.71, N 4.36; Found: C 88.65, H 6.92, N 4.43.

*Synthesis of 2,3-bis(4-(9,9-bis(6-(9H-carbazol-9-yl)hexyl)-9H-fluoren-2-yl)phenyl)fumaronitrile (trans-DFC)*: *trans*-DFC was synthesized similar as the preparation of SFC. Yield: 40%. ^1^H-NMR (500 MHz, CDCl_3_) δ 8.05 (dd, *J* = 20.2, 7.7 Hz, 8H), 7.97–7.91 (m, 4H), 7.80–7.75 (m, 5H), 7.72 (d, *J* = 7.4 Hz, 2H), 7.61 (dd, *J* = 7.8, 1.6 Hz, 2H), 7.54 (t, *J* = 5.9 Hz, 2H), 7.43–7.37 (m, 7H), 7.34 (td, *J* = 7.3, 1.2 Hz, 2H), 7.31–7.26 (m, 8H), 7.21–7.15 (m, 7H), 4.25–4.00 (m, 9H), 2.05–1.83 (m, 9H), 1.73–1.61 (m, 7H), 1.31–0.97 (m, 18H), 0.90–0.73 (m, 4H), 0.63 (qt, *J* = 13.9, 6.9 Hz, 8H). MALDI-TOF MS (mass *m/z*): 1557.2 [M+]. Anal. calcd. for C_114_H_102_N_6_: C 87.99, H 6.61, N 5.40; Found: C 88.20, H 6.45, N 5.35.

*cis-DFC Was obtained by exposure of trans-DFC to UV light for several hours. *^1^H-NMR (500 MHz, CDCl_3_) δ 8.06 (d, *J* = 7.7 Hz, 8H), 7.68 (dd, *J* = 12.4, 7.7 Hz, 4H), 7.58 (d, *J* = 8.6 Hz, 4H), 7.47 (dd, *J* = 11.0, 4.2 Hz, 8H), 7.41–7.35 (m, 8H), 7.34 (dd, *J* = 7.4, 1.0 Hz, 2H), 7.24 (t, *J* = 7.0 Hz, 9H), 7.21–7.16 (m, 8H), 4.11 (t, *J* = 7.2 Hz, 8H), 1.98–1.81 (m, 8H), 1.69–1.58 (m, 8H), 1.25–0.91 (m, 18H), 0.70–0.43 (m, 9H).

## 4. Conclusions

In summary, two AIE-active D-A-D compounds, SFC and DFC, are designed and synthesized by Suzuki coupling reactions. The pure *cis*- and *trans*-isomer of DFC were also successfully separated. All of them exhibit high quantum efficiencies in the solid state (η_F_ = 46% for SFC, 54.5% for *cis*-DFC, and 55.4% for *trans*-DFC) with good thermal stabilities and morphological stabilities. The introduction of one additional cyano group induced a big red-shift in solid-state emission in DFC, owing to the high electron-withdrawing ability. The *cis*- and *trans*-DFC show the similar absorption spectra, emission spectra, and CV behaviors. They can all be processed by low-cost spin-coating method to fabricated non-doped OLEDs. All three materials show identical EL spectra with their PL spectra. SFC showed green emission peaking at 472 nm with the highest EQE (1.37%), luminance (5201 cd m^−2^) and current efficiency (3.67 cd A^−1^). The *trans*-DFC showed an electroluminescence spectrum peaking at 576 nm with a maximum luminance of 4025 cd m^−2^, a maximum CE of 1.97 cd/A, and an EQE of 0.99%, which was a little higher than *cis*-DFC in terms of efficiencies (5716 cd m^−2^, 1.49 cd/A, 0.79%). The results pave a way for further understanding the structure–property relationship of diphenylfumaronitrile-based isomers.

## Figures and Tables

**Table 1:** Thermal, photophysical, and electrochemical data of SFC, *trans*-DFC, and *cis*-DFC.

Compounds	*T*_d_/*T*_g_^a^ (°C)	HOMO/LUMO ^b^ (eV)	λ^abs^_max_^c^ (nm)	λ^em^_max_^d^ (nm)	φ_film/powder_^e^ (%)
SFC	442/94	−5.48/−2.97	346/371	--/513	51/46
*trans*-DFC	438/90	−5.48/−3.51	400/426	570/587	40/54
*cis*-DFC	435/90	−5.48/−3.52	387/406	568/600	44/55

^a^*T*_d_ = thermal-decomposition temperature; *T*_g_ = glass-transition temperature; ^b^ Measured by cyclic voltammetry; ^c^ λ^abs^_max_ = maximum absorption wavelength in dilute tetrahydrofuran(THF) solution and spin-coated films; ^d^ λ^em^_max_ = maximum emission wavelength in dilute THF solution and spin-coated films; ^e^ Measured by integrating sphere (±1%).

**Table 2:** EL data of SFC, *trans*-DFC, and *cis*-DFC.

Compounds	Von ^a^ (V)	L_max_^b^ (cd m^−2^)	LE_max_^c^ (cd A^−1^)	λ^EL^_max_ (nm)	EQE ^d^ (%)	CIE (x, y)
SFC	5.0	5201	3.67	536	1.37	(0.36,0.52)
*trans*-DFC	5.0	4025	1.97	576	0.99	(0.49,0.50)
*cis*-DFC	6.0	5716	1.49	584	0.79	(0.51,0.48)

^a^ Von = turn on voltage; ^b^ L_max_ = the maximum luminance; ^c^ LE_max_ = the maximum luminance efficiency; ^d^ EQE = the maximum external quantum efficiency.

## Supplementary Materials

Supplementary materials are available online.

## References

[1] Tang C.W., VanSlyke S.A. (1987). Organic electroluminescent diodes. Appl. Phys. Lett..

[2] Yeh H.-C., Chan L.-H., Wu W.-C., Chen C.-T. (2004). Non-doped red organic light-emitting diodes. J. Mater. Chem..

[3] Wu K.C., Ku P.J., Lin C.S., Shih H.T., Wu F.I., Huang M.J., Lin J.J., Chen I.C., Cheng C.H. (2008). The Photophysical Properties of Dipyrenylbenzenes and Their Application as Exceedingly Efficient Blue Emitters for Electroluminescent Devices. Adv. Funct. Mater..

[4] Im Y., Byun S.Y., Kim J.H., Lee D.R., Oh C.S., Yook K.S., Lee J.Y. (2017). Recent Progress in High-Efficiency Blue-Light-Emitting Materials for Organic Light-Emitting Diodes. Adv. Funct. Mater..

[5] Nikolka M., Nasrallah I., Rose B., Ravva M.K., Broch K., Sadhanala A., Harkin D., Charmet J., Hurhangee M., Brown A. (2017). High operational and environmental stability of high-mobility conjugated polymer field-effect transistors through the use of molecular additives. Nat. Mater..

[6] Zhao Y., Guo Y., Liu Y. (2013). 25th anniversary article: Recent advances in n-type and ambipolar organic field-effect transistors. Adv. Mater..

[7] Lei T., Cao Y., Fan Y., Liu C.J., Yuan S.C., Pei J. (2011). High-performance air-stable organic field-effect transistors: Isoindigo-based conjugated polymers. J. Am. Chem. Soc..

[8] Zhang W., Han Y., Zhu X., Fei Z., Feng Y., Treat N.D., Faber H., Stingelin N., McCulloch I., Anthopoulos T.D. (2016). A Novel Alkylated Indacenodithieno[3,2-b]thiophene-Based Polymer for High-Performance Field-Effect Transistors. Adv. Mater..

[9] Rodríguez M., Ramos-Ortíz G., Alcalá-Salas M.I., Maldonado J.L., López-Varela K.A., López Y., Domínguez O., Meneses-Nava M.A., Barbosa-García O., Santillan R. (2010). One-pot synthesis and characterization of novel boronates for the growth of single crystals with nonlinear optical properties. Dyes Pigments.

[10] Li J., Yim D., Jang W.D., Yoon J. (2017). Recent progress in the design and applications of fluorescence probes containing crown ethers. Chem. Soc. Rev..

[11] Lee S.Y., Yasuda T., Komiyama H., Lee J., Adachi C. (2016). Thermally Activated Delayed Fluorescence Polymers for Efficient Solution-Processed Organic Light-Emitting Diodes. Adv. Mater..

[12] Birks J.B. (1970). Photophysics of Aromatic Molecules.

[13] Luo J., Xie Z., Lam J.W.Y., Cheng L., Tang B.Z., Chen H., Qiu C., Kwok H.S., Zhan X., Liu Y. (2001). Aggregation-induced emission of 1-methyl-1,2,3,4,5-pentaphenylsilole. Chem. Commun..

[14] Mei J., Leung N.L., Kwok R.T., Lam J.W., Tang B.Z. (2015). Aggregation-Induced Emission: Together We Shine, United We Soar!. Chem. Rev..

[15] Yang Z., Qin W., Leung N.L.C., Arseneault M., Lam J.W.Y., Liang G., Sung H.H.Y., Williams I.D., Tang B.Z. (2016). A mechanistic study of AIE processes of TPE luminogens: Intramolecular rotation vs. configurational isomerization. J. Mater. Chem. C.

[16] Mei J., Hong Y., Lam J.W., Qin A., Tang Y., Tang B.Z. (2014). Aggregation-induced emission: The whole is more brilliant than the parts. Adv. Mater..

[17] Kwok R.T., Leung C.W., Lam J.W., Tang B.Z. (2015). Biosensing by luminogens with aggregation-induced emission characteristics. Chem. Soc. Rev..

[18] Han X., Bai Q., Yao L., Liu H., Gao Y., Li J., Liu L., Liu Y., Li X., Lu P. (2015). Highly Efficient Solid-State Near-Infrared Emitting Material Based on Triphenylamine and Diphenylfumaronitrile with an EQE of 2.58% in Nondoped Organic Light-Emitting Diode. Adv. Funct. Mater..

[19] Tang S., Liu M.R., Lu P., Xia H., Li M., Xie Z.Q., Shen F.Z., Gu C., Wang H.P., Yang B. (2007). A Molecular Glass for Deep-Blue Organic Light-Emitting Diodes Comprising a 9,9′-Spirobifluorene Core and Peripheral Carbazole Groups. Adv. Funct. Mater..

[20] Liu H., Cheng G., Hu D., Shen F., Lv Y., Sun G., Yang B., Lu P., Ma Y. (2012). A Highly Efficient, Blue-Phosphorescent Device Based on a Wide-Bandgap Host/FIrpic: Rational Design of the Carbazole and Phosphine Oxide Moieties on Tetraphenylsilane. Adv. Funct. Mater..

[21] Yao L., Xue S., Wang Q., Dong W., Yang W., Wu H., Zhang M., Yang B., Ma Y. (2012). RGB small molecules based on a bipolar molecular design for highly efficient solution-processed single-layer OLEDs. Chem. Eur. J..

[22] Hong Y., Lam J.W., Tang B.Z. (2009). Aggregation-induced emission: Phenomenon, mechanism and applications. Chem. Commun..

[23] Ellinger S., Graham K.R., Shi P., Farley R.T., Steckler T.T., Brookins R.N., Taranekar P., Mei J., Padilha L.A., Ensley T.R. (2011). Donor–Acceptor–Donor-based π-Conjugated Oligomers for Nonlinear Optics and Near-IR Emission. Chem. Mater..

[24] Li J., Han X., Bai Q., Shan T., Lu P., Ma Y. (2017). Electropolymerized AIE-active polymer film with high quantum efficiency and its application in OLED. J. Polym. Sci. Part A Polym. Chem..

[25] Gu C., Huang N., Wu Y., Xu H., Jiang D. (2015). Design of Highly Photofunctional Porous Polymer Films with Controlled Thickness and Prominent Microporosity. Angew. Chem. Int. Ed. Engl..

