# Supplementary material for: Preparation of Cyano-Substituted Tetraphenylethylene Derivatives and Their Applications in Solution-Processable OLEDs
Source: Molecules. 2018 Jan 17;23(1):190. doi: 10.3390/molecules23010190 (PMC6017102; doi:10.3390/molecules23010190)
Supplement: Supplementary file 1 [file molecules-23-00190-s001.pdf]

Supporting Materials

Preparation of Cyano-Substituted Tetraphenylethylene Derivatives and Their Applications in Solution-Processable OLEDs

Xiaoyi Sun [1], Lele Zhao [1,2], Xiao Han [1], Hui Liu [1], Yu Gao [1], Yanchun Tao [1], Haiquan Zhang [2], Bing Yang [1] and Ping Lu [1,*]

[1] State Key Laboratory of Supramolecular Structure and Materials, Jilin University, 2699 Qianjin Avenue, Changchun 130012, China; xysun15@mails.jlu.edu.cn (X.S.); challenge_llzhao@163.com (Z.L.); slan521241@126.com (X.H.); liuhui17@mails.jlu.edu.cn (H.L.); iambird83317035@126.com (Y.G.); taoyc@jlu.edu.cn (Y.C.); yangbing@jlu.edu.cn (Y.B.); lup@jlu.edu.cn (L.P.)

[2] State Key Laboratory of Metastable Materials Science and Technology, Yanshan University, Qinhuangdao 066004, China; hqzhang@ysu.edu.cn (H.Z.)

* Correspondence: lup@jlu.edu.cn

Index

Figure S1. (A) PL spectra of SFC in THF-water mixtures with different water fractions (f_w). (B) Plot of the relative PL intensity versus the compositions of THF-water mixtures of SFC

Figure S2. (A) PL spectra of cis-DFC in THF-water mixtures with different water fractions (f_w). (B) Plot of the relative PL intensity (I/I_0) versus the compositions of THF-water mixtures of cis-DFC. I_0 = emission intensity in pure THF solution. Inset in (B) are the fluorescent photographs at f_w = 0 and 95% taken under a 365 nm UV lamp.

Figure S3. ^{1}H NMR spectra of trans-DFC and cis-DFC in $CDCl_3$ (500 M Hz, 298 K).

Figure S4. The EL spectra of devices of SFC, trans-DFC and cis-DFC under the different operating voltages.

Figure S5. (A) PL spectra of trans-DFC in different solvents (10^{-5} M); (B) PL spectra of cis-DFC in different solvents (10^{-5} M).

Figure S6. X-ray diffraction (XRD) patterns of SFC, trans-DFC and cis-DFC in powder state.

Figure S7. TEM images (left) and ED patterns (right) of (A and B) amorphous and (C and D) crystalline aggregates of trans-DFC formed in the acetonitrile/water mixtures with water contents of 0 and 90 vol %.

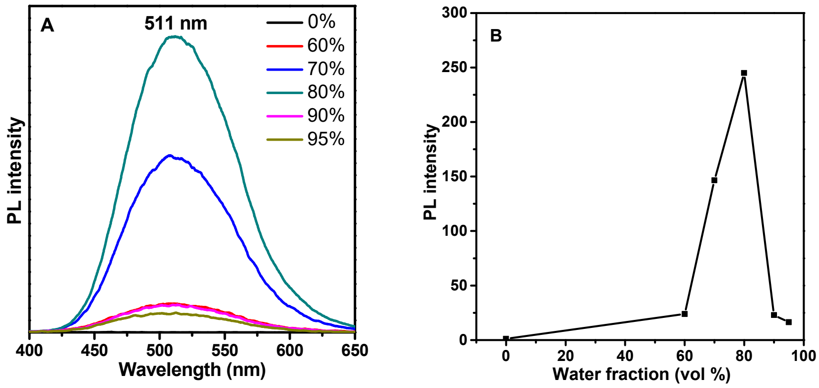

Figure S1. (A) PL spectra of SFC in THF-water mixtures with different water fractions (f_w). (B) Plot of the relative PL intensity versus the compositions of THF-water mixtures of SFC.

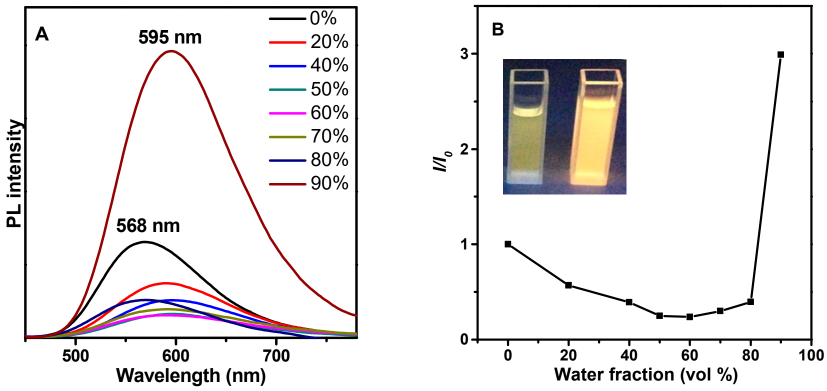

Figure S2. (A) PL spectra of cis-DFC in THF-water mixtures with different water fractions (f_w). (B) Plot of the relative PL intensity (I/I_0) versus the compositions of THF-water mixtures of cis-DFC. I_0 = emission intensity in pure THF solution. Inset in (B) are the fluorescent photographs at f_w = 0 and 95% taken under a 365 nm UV lamp.

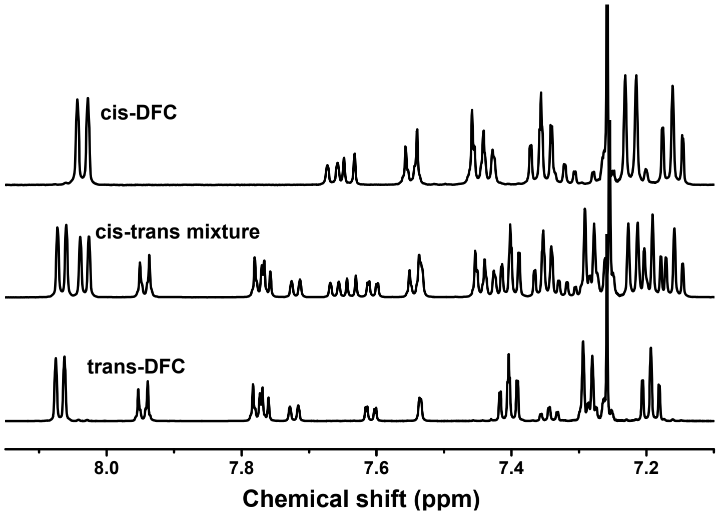

Figure S3. ^{1}H NMR spectra of trans-DFC and cis-DFC in $CDCl_3$ (500 M Hz, 298 K).

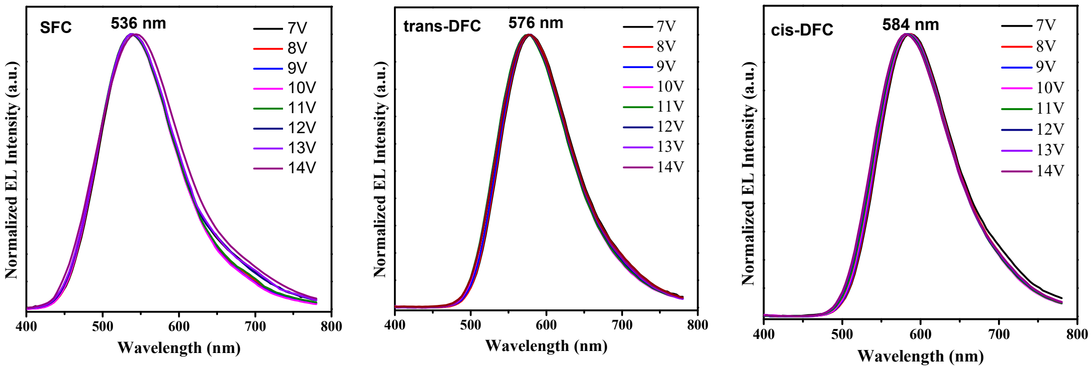

Figure S4.The EL spectra of devices of SFC, trans-DFC and cis-DFC under the different operating voltages

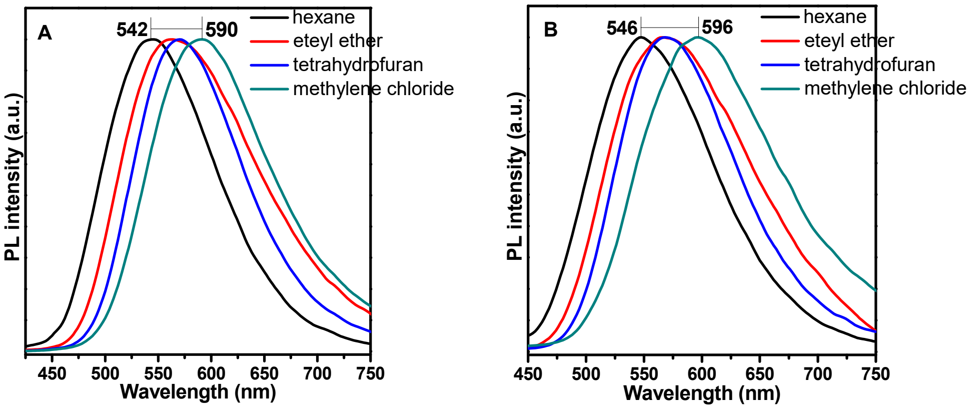

Figure S5. (A) PL spectra of trans-DFC in different solvents (10^{-5} M); (B) PL spectra of cis-DFC in different solvents (10^{-5} M).

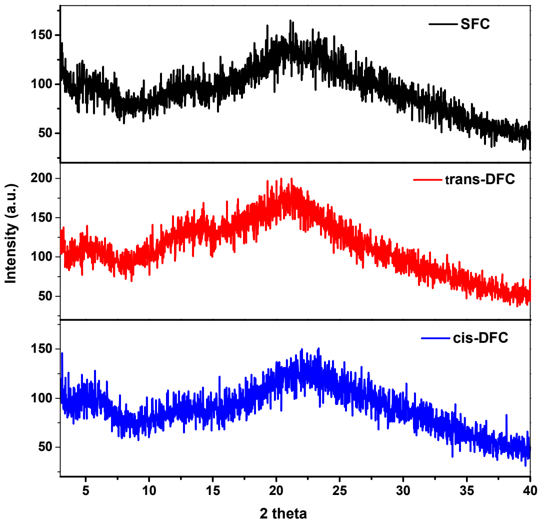

Figure S6. X-ray diffraction (XRD) patterns of SFC, trans-DFC and cis-DFC in powder state.

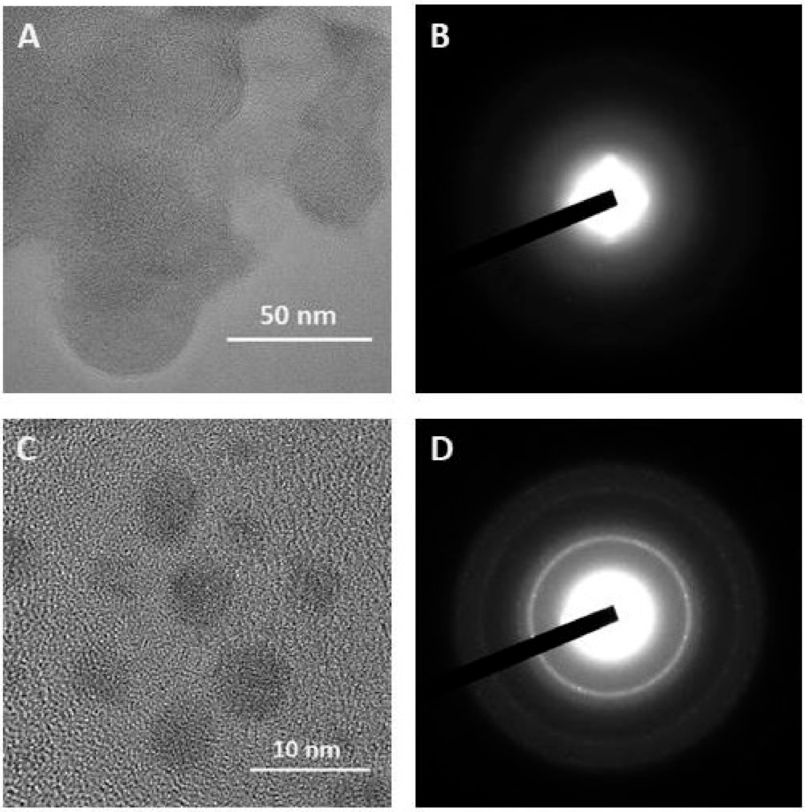

44 **Figure S7.** TEM images (left) and ED patterns (right) of (A and B) amorphous and (C and D)
45 crystalline aggregates of trans-DFC formed in the acetonitrile/water mixtures with water contents of
46 0 and 90 vol %.
